# Medical-nursing integrated service model in ophthalmic outpatient care for intravitreal injections: a retrospective cohort study

**DOI:** 10.3389/fmed.2025.1689049

**Published:** 2025-12-16

**Authors:** Genyin Lan, Zhuoxin Li, Wenhui Cheng, Ruiyu Huang, Yanxia Guo, Xiaofang Yang, Baolu Zhang

**Affiliations:** 1 Department of Ophthalmology, The Affiliated Hospital, Southwest Medical University, Luzhou, China; 2 Department of Nursing, The Affiliated Hospital, Southwest Medical University, Luzhou, China; 3 School of Nursing, Southwest Medical University, Luzhou, China; 4 School of Continuing Education, Guiyang Healthcare Vocational University, Guiyang, China; 5 Faculty of Nursing and Midwifery, Jiangsu College of Nursing, Huaian, China; 6 Guiyang Maternal and Child Health Care Hospital, Guiyang, China

**Keywords:** ophthalmology, medical-nursing integrated service model, outpatient care, intravitreal injection, quality improvement

## Abstract

**Background:**

Outpatient intravitreal injections are widely utilized in contemporary ophthalmology practice. However, fragmented medical-nursing coordination and compressed clinical pathways challenge treatment standardization and compromise patient outcomes. This study aimed to evaluate the effectiveness of an integrated medical-nursing service model compared to conventional separated care in patients undergoing outpatient intravitreal injections.

**Methods:**

A retrospective cohort study was conducted involving 1,432 patients undergoing outpatient intravitreal injections at our hospital's ophthalmology department. Patients were divided into two groups: a control group (*n* = 631) receiving conventional separated medical and nursing care and an intervention group (*n* = 801) experiencing an integrated medical-nursing service model. Key outcome measures included patient satisfaction, correct usage rates of preoperative antibiotic eye drops, standardized treatment rates, vision-related quality of life using NEI-VFQ-25 scores, and complication rates.

**Results:**

Compared with the control group, the intervention group showed significantly higher patient satisfaction (*P* < 0.001), higher correct usage rates of preoperative antibiotic eye drops (*P* < 0.001), higher standardized treatment rates (*P* < 0.001), and improved NEI-VFQ-25 scores (*P* < 0.001). Additionally, the intervention group experienced significantly lower rates of complications, including subconjunctival hemorrhage (1.12% vs. 5.71%), postoperative intraocular pressure elevation (0.25% vs. 3.33%), and infectious conjunctivitis (0.50% vs. 3.65%), all with *P* < 0.001.

**Conclusions:**

The medical-nursing integrated service model significantly improves patient satisfaction, medication compliance, standardized treatment rates, and vision-related quality of life and reduces complications in outpatient intravitreal injections procedures. These findings suggest that this model effectively addresses key challenges in outpatient procedure delivery while enhancing both clinical outcomes and patient experience.

## 1 Introduction

Ophthalmology, as a specialized medical field, is characterized by precise microsurgical procedures, standardized surgical protocols, and relatively low surgical risks. This is evidenced in the widespread adoption of minimally invasive techniques across various methods, such as Minimally Invasive Glaucoma Surgery (MIGS), small-incision cataract surgery, and intravitreal injections (1–3).

In China, ocular diseases represent a substantial healthcare concern, with a considerable number of patients (over 70 million) affected by major eye conditions. Epidemiological data reveal 31.58 million patients with diabetic retinopathy (2021 International Diabetes Federation statistics), 9.4 million with diabetic macular edema, 26.65 million with age-related macular degeneration (2015 data), and 6.85 million with retinal vein occlusion (4, 5). The severity of these conditions frequently contributes to irreversible visual function loss, which can significantly affect patients' quality of life (6, 7). Global statistics from 2020 provide evidence that among individuals aged 50 and above, 1.8 million are affected by AMD, with 6.2 million experiencing moderate to severe visual impairment. Epidemiological projections suggest a potential increase to 288 million cases by 2040 (8).

In the Chinese healthcare context, the management of these retinal diseases faces several system-level challenges. While anti-VEGF drugs have been gradually included in medical insurance coverage since 2017, patients still face substantial out-of-pocket expenses. Optical coherence tomography (OCT) equipment availability varies between different hospital levels, and treatment waiting times in major centers can extend to several weeks. These factors underscore the need for more efficient care delivery models to improve patient access and treatment outcomes.

Faced with these ophthalmological challenges, improving healthcare service efficiency becomes crucial. The outpatient care model offers an effective approach to addressing the treatment needs of the large volume of ophthalmological patients by streamlining hospitalization processes. Day-case care represented a transformation from traditional 24-h hospitalization to a streamlined process involving day admission, treatment, and discharge while maintaining optimal standards of medical quality and safety (9). Ophthalmic surgeries, particularly those classified as levels 1–3 according to our institutional surgical complexity grading system (based on procedure invasiveness, duration, and perioperative risk profile), are highly suitable for this model due to their minimal invasiveness and relatively low-risk profile. Level 1 procedures include chalazion excision, pterygium removal, and minor eyelid surgeries; Level 2 encompasses cataract extraction, trabeculectomy, and vitrectomy for uncomplicated cases; Level 3 includes complex retinal procedures, corneal transplantation, and orbital surgeries. While this approach has gained significant attention in ophthalmology due to its minimally invasive nature and rapid recovery potential, outpatient intravitreal injection services require meticulous attention to patient management and monitoring to mitigate risks associated with shortened hospital stays and increased patient turnover. The substantial economic burden imposed by eye diseases underscores the importance of such efficient healthcare service models, as both direct medical expenses and indirect costs pose significant financial challenges for patients and healthcare systems.

To address both clinical demands and economic pressures, efficient care delivery models have become critical. While 6–8 h same-day service models have become increasingly prevalent in major Chinese ophthalmology centers, implementation remains heterogeneous across institutions. At our institution in 2023, the standard pathway for intravitreal injections followed a 24-h hospitalization model due to existing institutional protocols.

A study highlighted the advantages of outpatient care in healthcare settings, which is particularly relevant for ophthalmology with its standardized procedures and established safety protocols (10). These benefits include reduced risk of hospital-acquired infections, facilitated patient recovery in familiar environments, and optimized utilization of hospital resources. In ophthalmology, where procedures are predominantly minimally invasive and highly standardized, these advantages can be effectively realized while maintaining high standards of care. However, successful implementation requires careful attention to preoperative preparation, efficient perioperative management, and comprehensive post-discharge care instructions.

The medical-nursing integrated service model, as part of China's healthcare reform initiatives, represented a practical approach to enhancing coordination between medical and nursing services (11). Previous studies have explored various integrated care models in ophthalmology and other surgical specialties, with research by international healthcare systems demonstrating benefits of collaborative care approaches in ambulatory settings (12). Studies examining nurse-delivered intravitreal injection services have shown enhanced treatment capacity while maintaining patient safety and satisfaction (13, 14), while physician-nurse collaboration has been associated with improved patient safety culture and outcomes across healthcare settings (15). However, systematic evaluation of comprehensive medical-nursing integrated models specifically for intravitreal injection procedures in ophthalmology settings remains limited.

Theoretical frameworks from quality improvement science, particularly Donabedian's Structure-Process-Outcome model, support systematic care delivery modifications to enhance patient outcomes (16). While fragmented care delivery remains common in traditional hospital settings, integrated care models have demonstrated superior outcomes across diverse healthcare contexts (17). Originating from ongoing developments in China's healthcare system, this model aims to optimize collaboration between medical and nursing teams to address patients' healthcare needs and improve service quality (18).

In ophthalmology departments, where common challenges include high patient volumes, substantial surgical loads, bed shortages, and admission difficulties, the medical-nursing integrated service model can play a crucial role. This was particularly relevant given the large patient population requiring treatment and the significant economic burden associated with eye diseases, as evidenced by cost analysis data from various Chinese cities showing disparities in medical expenses and treatment costs.

Although same-day discharge addresses efficiency concerns, coordination challenges persist across different care models. Even in established 6–8 h same-day services, common limitations include fragmented medical-nursing communication, inconsistent patient education, variable compliance monitoring, and discontinuous follow-up. Our integrated model's innovation lies in systematically restructuring care coordination through dedicated interprofessional teams, structured handover protocols, real-time communication platforms, and hierarchical follow-up systems. This approach addresses fundamental coordination deficits rather than merely shortening hospitalization duration, offering transferable principles applicable to institutions at various developmental stages, including those with existing same-day services seeking to optimize team collaboration.

Against this backdrop, our research examined the implementation of the medical-nursing integrated service model in ophthalmic outpatient intravitreal injections, with a specific focus on intravitreal injections. This study aimed to comprehensively evaluate the model's effectiveness through multiple outcome measures: patient satisfaction, correct usage rates of preoperative antibiotic eye drops, standardized treatment rates, vision-related quality of life, and complication incidence. By assessing these parameters, we sought to provide evidence-based insights into optimizing care delivery for the growing population of patients requiring ophthalmic interventions.

## 2 Materials and methods

### 2.1 Study design

This retrospective cohort study included data from 1,432 patients who underwent intravitreal injections as day surgery at our hospital's ophthalmology department. We employed a before-and-after study design to evaluate the implementation of the medical-nursing integrated service model. The control group comprised 631 patients who received conventional separated care from January 1, 2023 to December 31, 2023, while the intervention group included 801 patients who received the integrated medical-nursing service model from January 1, 2024 to December 31, 2024. As a retrospective observational study, this research was not prospectively registered in a clinical trial registry in accordance with ICMJE guidelines. The integrated service model was officially implemented on January 1, 2024, following a 2-month preparation period including staff training and workflow optimization in late 2023.

### 2.2 Study hypothesis

We hypothesized that compared to conventional separated medical and nursing care, the integrated medical-nursing service model would significantly improve multiple clinical outcomes in patients undergoing outpatient intravitreal injections. Specifically, we hypothesized that the integrated model would: (1) increase the correct usage rates of preoperative antibiotic eye drops, (2) improve standardized treatment rates (defined as patients receiving ≥3 intravitreal injections), (3) enhance vision-related quality of life as measured by NEI-VFQ-25 scores, (4) reduce postoperative complication rates, and (5) improve overall patient satisfaction with nursing care.

### 2.3 Patient selection

#### 2.3.1 Inclusion criteria

(1) Patients undergoing outpatient ophthalmological intravitreal injection. (2) Patients diagnosed with one of the following four conditions: Wet age-related macular degeneration (WAMD), Diabetic macular edema (DME), Macular edema secondary to retinal vein occlusion (RVO, ME), Choroidal neovascularization (CNV). (3) Patients with relatively stable general health conditions. (4) Patients who provide informed consent.

#### 2.3.2 Exclusion criteria

(1) Patients with severe cardiovascular or cerebrovascular diseases. (2) Patients with severe hepatic or renal insufficiency. (3) Patients with serious malignant tumors. (4) Patients with a history of psychiatric disorders or inability to communicate normally. (5) Patients with severe immune system disorders or coagulation dysfunction.

#### 2.3.3 Diagnostic procedures and assessments

All patients underwent standardized diagnostic evaluations prior to intravitreal injection:

Standard Preoperative Examinations: (1) Visual acuity assessment using standardized charts. (2) Intraocular pressure measurement with non-contact tonometry. (3) Slit-lamp biomicroscopy for anterior segment evaluation. (4) Fundus examination with indirect ophthalmoscopy. (5) Optical coherence tomography (OCT) for macular assessment and treatment monitoring.

Selective Corneal Topography: Corneal topography was performed selectively based on clinical indication, primarily for: (1) Patients with history of corneal disease or previous corneal surgery. (2) Cases where corneal irregularities might affect injection site selection. (3) Patients reporting visual disturbances potentially related to corneal pathology. (5) Documentation of baseline corneal status in complex cases.

Supplementary Clinical Assessments: (1) Fluorescein angiography when clinically indicated for treatment planning. (2) Blood pressure and blood glucose monitoring for diabetic patients. (3) Review of current medications and anticoagulant therapy status.

Assessment Standardization: All diagnostic procedures followed standardized protocols with consistent equipment calibration and operator training to ensure reproducibility and reliability of measurements across both study groups. The same clinical criteria and examination standards were applied uniformly regardless of group assignment to maintain diagnostic consistency throughout the study period.

#### 2.3.4 Treatment workflow and timeline

**Figure 1:**
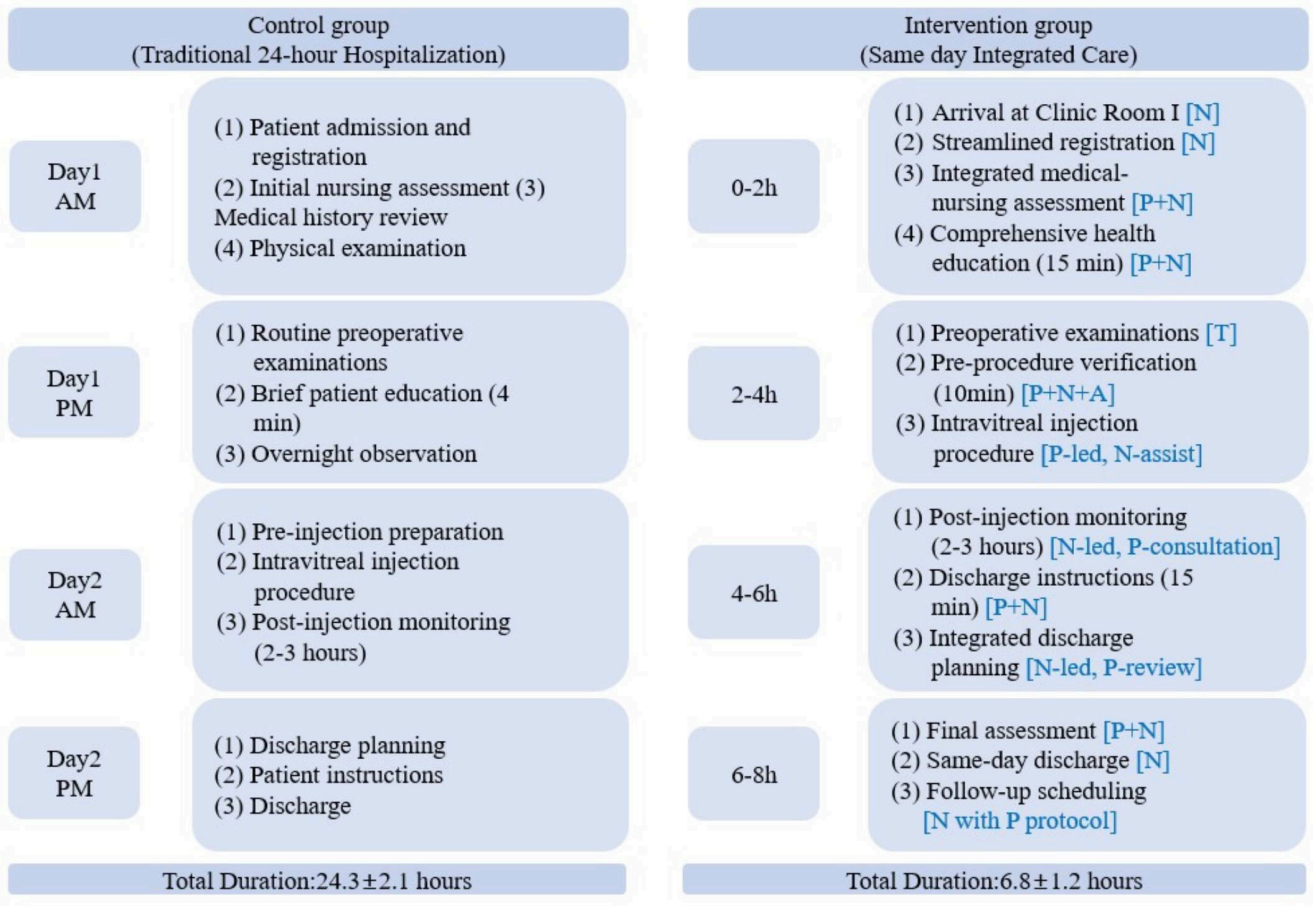
Flowchart comparing the two modes. P, Physician led; N, Nurse led; T, Technician; A, Anesthesiologist; P+N, collaborative.

Both groups underwent initial assessment and intravitreal injection procedures on the same treatment day, with distinct care delivery models. The control group followed a traditional 24-h hospitalization pathway, while the intervention group utilized a streamlined same-day approach within 6–8 h (Figure 1, Table 1).

**Table 1:** Operational characteristics comparison between care models.

**Characteristic**	**Control group (fragmented care)**	**Intervention group (integrated care)**
Team structure	Rotating staff, no fixed assignments	Fixed team: 1 chief physician + 1 associate chief physician + 2 attending physicians + 2 nurses + 2 technicians + 1 anesthesiologist
Physical environment	Standard shared ward	Dedicated Clinic Room 1 with specialized equipment (slit lamp, tonometer, corneal topographer, multimedia education system)
Patient education method	Brief verbal instruction (4 min) Physician and nurse separately	Structured session (15 min) Multimedia-enhanced Joint physician-nurse delivery
Communication system	Written orders + verbal handover	Electronic platform + real-time WeChat group with instant messaging and photo documentation
Pre-procedure verification	Physician checklist only	Collaborative triple-check (Physician + Nurse + Anesthesiologist)
Post-discharge follow-up	Optional telephone contact Next-day ward round Subsequent contact optional	Mandatory structured protocol: Day 1: Telephone (nurse-led, 20 min) Day 7: In-person (mandatory) Day 30: Comprehensive evaluation Real-time: WeChat (< 2 h response)
Quality control	General hospital standards *Ad hoc* Review	Procedure-specific monitoring Monthly team reviews Quarterly emergency drills Real-time complication tracking
Emergency response	General hospital protocol Variable response time	Dedicated 24/7 on-call system < 15-min response time Protocol-specific action plans Pre-positioned emergency equipment
Patient stay duration	24.3 ± 2.1 h (overnight hospitalization)	6.8 ± 1.2 h (same-day discharge)

The control group required overnight observation with standard ward-based care, whereas the intervention group achieved same-day discharge through dedicated clinical space and integrated medical-nursing team coordination. Both groups maintained identical clinical standards for patient assessment and injection procedures. The fundamental differences in care delivery approaches are illustrated in Figure 1 and detailed in Table 1.

### 2.4 Intervention procedures

Control group: The control group received conventional care services within the standard hospital protocol. Upon admission, ward nurses managed admission procedures and introduced hospital regulations and the environment to patients and their families. After bed assignment, nurses notified attending physicians, who then conducted routine history-taking and physical examinations before prescribing treatment plans. Responsible nurses implemented medical orders, provided routine care, delivered patient education about disease and surgery, offered discharge guidance, and provided psychological support throughout the patient's stay.

Intervention group: The intervention group adopted a medical-nursing integrated service model with ten interconnected components implemented simultaneously:

1. Establishment of outpatient care team: An outpatient care team was established as the foundation for conducting outpatient procedures. The director (responsible for establishing treatment indication criteria and reviewing treatment plans) and head nurse (responsible for setting nursing quality standards and personnel training plans) served as primary responsible persons. The operational team consisted of one chief physician, one associate chief physician, two attending physicians (all qualified for independent procedures), two nurses (with over 5 years of ophthalmology nursing experience), two technicians (responsible for preoperative examinations), and one anesthesiologist (fixed scheduling). As part of our integrated care model, a fixed anesthesiologist was assigned to ensure comprehensive patient safety monitoring and emergency preparedness, rather than for routine anesthesia administration.
2. Designated clinic room: Outpatient Clinic Room one was specifically designated for receiving and treating outpatient patients. After initial patient assessment, if the outpatient doctor determined the patient was suitable for outpatient treatment, they issued a guidance form (specifying eye and procedure) directing the patient to report to Clinic Room one. The clinic room was equipped with professional examination equipment (slit lamp, tonometer, corneal topographer, etc.), an independent health education area with multimedia educational equipment, standardized assessment tools, and information technology equipment for medical-nursing collaboration.
3. Clinic Room one had two permanent doctors and two nurses with a shift system and backup mechanism in place, along with detailed job descriptions and performance evaluation systems. Doctors mainly handled patient communication, prescribed preoperative examinations, reviewed examination reports, scheduled procedures, obtained consent signatures, and conducted follow-up visits. Nurses were primarily responsible for recording patient basic information, guiding patients through preoperative examinations, preliminary review of examination completion, and distributing outpatient procedure patient instructions.
4. Medical-nursing team collaboration: After doctors explain patient conditions, nurses can assist in addressing any remaining patient concerns, increasing doctors' effective working time. After nurses provide health education, doctors can reinforce key points, with both medical and nursing staff jointly answering questions, complementing and strengthening each other's efforts to meet patient needs and ensure safety. The team establishes daily joint ward rounds and regular team meetings, developing standardized collaborative work forms.
5. Establishing medical-nursing integrated handover system: Medical and nursing staff jointly develop handover systems and requirements, establishing integrated medical-nursing handover records with standardized handover checklists, specific handover time points, handover quality evaluation, and electronic handover platform. Detailed handovers of outpatient procedure patients' conditions and special circumstances ensure team members comprehensively understand each patient's specific situation, providing continuous and comprehensive medical-nursing care. Additionally, an integrated medical-nursing WeChat group facilitates real-time communication about work-related issues, mutual reminders and support, discussion of challenging cases, and sharing of successful cases and experiences for mutual improvement.
6. Establishing medical-nursing integrated follow-up system: Upon discharge, nurses create patient files and develop hierarchical follow-up plans based on medical orders and specific patient conditions. Standard follow-up forms and quality control indicators are established. Follow-up methods include telephone and WeChat communication. During telephone follow-ups, doctors promptly address any medical questions that arise. Patients can consult through the WeChat group about various issues after returning home, receiving guidance from both medical and nursing perspectives. Regular and irregular dissemination of disease-related educational content and follow-up reminders is provided.
7. Quality control system: A comprehensive quality monitoring framework tracked treatment processes and patient outcomes. Standardized complication prevention checklists were implemented and verified before each procedure. All adverse events were documented using standardized reporting forms with immediate notification protocols. Monthly quality improvement meetings systematically reviewed clinical outcomes, workflow efficiency, and patient feedback, with root cause analysis conducted for complications. Medical-nursing joint quality evaluations assessed collaborative care delivery and identified areas for improvement.
8. Information support system: A specialized electronic platform facilitated real-time care coordination among team members. The system provided: (a) centralized patient status tracking accessible to physicians, nurses, and technicians, (b) automated notifications at key care transition points to ensure timely handovers, (c) structured communication channels for case discussions and clinical decision-making, (d) automated appointment reminders delivered via SMS and WeChat to enhance follow-up adherence, and (e) electronic documentation of quality monitoring activities. All staff completed training on system operation before clinical implementation.
9. Emergency response management: Pre-positioned emergency equipment and medications were maintained in the procedure room, including IOP-lowering agents, anterior chamber paracentesis supplies, and endophthalmitis treatment materials. Detailed escalation protocols specified criteria for activating specialist consultations when complications exceeded standardized management thresholds. A 24-h on-call physician and nurse system ensured continuous availability for telephone consultation and urgent in-person evaluation. Quarterly emergency simulation drills tested team responses to various complication scenarios, ensuring procedural familiarity and coordination.
10. Continuous improvement mechanism: Monthly team meetings systematically reviewed clinical outcomes, workflow challenges, and coordination issues, with specific improvement initiatives assigned to responsible team members. Staff engagement and satisfaction were assessed through quarterly structured surveys. Patient feedback was actively solicited through multiple channels, including post-procedure questionnaires, follow-up telephone contacts, and physical suggestion boxes in the clinic area. A rapid-cycle improvement approach enabled prompt implementation of workflow refinements, with substantive changes undergoing pilot evaluation before broader adoption.

All ten components were fully implemented and maintained operational throughout the 2024 study period. Prior to implementation, comprehensive staff training was completed covering standardized clinical procedures, communication protocols, emergency response algorithms, and quality documentation requirements. Ongoing compliance monitoring through regular team audits verified sustained implementation of all system components.

### 2.5 Implementation specifications and operational metrics

#### 2.5.1 Care duration and staffing

The intervention group achieved same-day discharge with mean stays of 6.8 ± 1.2 h compared to the control group's 24.3 ± 2.1 h overnight hospitalization. The intervention utilized a dedicated team comprising one chief physician, one associate chief physician, two attending physicians (8-h shifts), two specialized nurses (12-h shifts), two technicians, and one anesthesiologist.

#### 2.5.2 Educational and communication protocols

Patient education differed substantially between groups: the intervention group received structured 15-min sessions incorporating disease-specific education, comprehensive procedural instructions, and multimedia demonstrations, while the control group received brief 4-min verbal education with standard written materials. The intervention group followed a standardized communication framework with three touchpoints: initial consultation (20 min), pre-procedure verification (10 min), and post-procedure discharge planning (15 min).

#### 2.5.3 Follow-up implementation

The intervention group utilized structured post-discharge monitoring with three mandatory touchpoints: Day 1 nurse-led telephone assessment (20 min, standardized symptom inquiry and medication compliance verification), Day 7 mandatory ophthalmologist examination (visual acuity, intraocular pressure, slit-lamp and fundus examination), and Day 30 comprehensive ophthalmologist evaluation (including OCT imaging and treatment plan adjustment). Automated reminders (SMS/WeChat) were sent 24–48 h before scheduled visits to ensure attendance. Real-time WeChat consultation was available with response times under 2 h during business hours. Control group received next-day physician ward rounds during hospitalization, followed by optional outpatient follow-up without structured protocols.

### 2.6 Outcome measures

#### 2.6.1 Preoperative antibiotic eye drops compliance rate

This was calculated as the number of patients who correctly administered antibiotic eye drops according to medical instructions before surgery divided by the total number of surgical patients × 100%. Following the 2015 Chinese consensus guidelines for intravitreal injection quality control (19), all patients receiving intravitreal injections received topical levofloxacin 0.5% four times daily for 3 days prior to injection. We acknowledge that this practice differs from current international recommendations, which generally do not recommend routine antibiotic prophylaxis due to limited evidence of efficacy (20). However, adherence to institutional protocols was maintained throughout the study period to ensure consistency.

#### 2.6.2 Rate of standardized treatment of patients

Based on previous research findings, three or more intravitreal injections are considered a standardized treatment (21). Standardized treatment was defined according to the “3+PRN” regimen recommended by Chinese clinical guidelines for diabetic retinopathy, retinal vein occlusion, and age-related macular degeneration, which specify a minimum of three initial monthly injections (22–24). Patients receiving ≥3 injections were classified as the standardized treatment group, while those receiving < 3 injections were classified as the non-standardized treatment group.

#### 2.6.3 NEI-VFQ-25 score

The National Eye Institute Visual Function Questionnaire-25 (NEI-VFQ-25) was administered to patients in both groups at baseline (before treatment) and at the final follow-up visit to assess changes in visual function-related quality of life. This validated instrument evaluates 12 domains of vision-related function, including general vision, near and distance vision activities, peripheral vision, color vision, ocular pain, driving difficulties, vision-specific social functioning, mental health, role difficulties, and dependency. The questionnaire generated a composite score ranging from 0 to 100, with higher scores indicating better visual function and quality of life. The Chinese version of NEI-VFQ-25, which had been validated for reliability and construct validity in previous studies (25), was used in this research.

#### 2.6.4 Complication rate

All postoperative complications were systematically documented and graded according to severity. Both major complications (endophthalmitis, retinal detachment, persistent vision loss, corneal injury requiring intervention) and minor complications (subconjunctival hemorrhage, transient intraocular pressure elevation, mild infectious conjunctivitis) were tracked. Structured follow-up protocols were implemented at 1 day, 1 week, and 1 month to detect both immediate and delayed-onset complications. Any vision-threatening complications or those requiring additional intervention were classified as serious adverse events and reported according to institutional safety protocols.

#### 2.6.5 Patient satisfaction

The patient satisfaction questionnaire was developed internally through a systematic process involving literature review, expert panel consultation (three senior ophthalmologists, two nursing administrators, one healthcare quality specialist), and pilot testing with 50 patients. Following initial development, the questionnaire was externally validated at two independent healthcare institutions with similar patient populations (*n* = 120 patients), demonstrating consistent psychometric properties across different settings. The 20-item instrument covers five domains: communication quality, care coordination, technical competence, facility environment, and overall experience, using a five-point Likert scale with a total possible score of 100 points.

Cut-off points were established based on institutional quality benchmarks and quartile distribution from pilot testing: very satisfied (≥80 points), satisfied (60–79 points), and dissatisfied ( ≤ 59 points). These thresholds were validated against patient willingness to recommend services (*r* = 0.76, *P* < 0.001). The overall satisfaction rate was calculated as (very satisfied + satisfied)/total cases × 100%. The questionnaire demonstrated acceptable internal consistency (Cronbach's α = 0.843) and content validity (Content Validity Index = 0.89). Survey Administration: Questionnaires were administered specifically for this research study by trained research nurses (not direct care providers) immediately prior to discharge. An anonymous collection with explicit confidentiality assurance was emphasized to encourage honest feedback regardless of cultural background.

### 2.7 Data collection

#### 2.7.1 Patient demographics and clinical data

Patient demographic data (age, gender) and clinical characteristics (diagnosis type, disease duration, previous treatment history) were extracted from electronic medical records by trained research staff using standardized data collection forms. All data extraction was performed by two independent reviewers with discrepancies resolved by consensus.

#### 2.7.2 Medication compliance evaluation

Preoperative antibiotic eye drop compliance was assessed through direct patient interview and medication diary review at the time of procedure. Patients were asked to demonstrate proper instillation technique and report adherence to prescribed frequency and duration.

#### 2.7.3 Visual function assessment

NEI-VFQ-25 questionnaires were administered by trained staff at baseline and follow-up visits. Responses were scored according to standard protocols, with higher scores indicating better visual function.

#### 2.7.4 Complication monitoring

To ensure comprehensive complication detection, all patients underwent mandatory structured follow-up at predetermined intervals (1 day, 1 week, and 1 month post-injection). Follow-up assessments included visual acuity measurement, intraocular pressure monitoring, anterior segment examination, and fundus evaluation.

IOP was measured using a standardized protocol with a non-contact tonometer (NCT) by the same trained technician using the same instrument throughout the study. Three consecutive measurements were taken and averaged; if the difference exceeded 2 mmHg, additional measurements were performed until stable values were obtained. Measurement timing differed between groups: intervention group patients had IOP measured 2 h before injection, 30 min post-injection, and 1 h post-injection; control group patients had IOP measured at presentation and on the morning of the second day post-injection. Ocular hypertension was defined as IOP >21 mmHg, with immediate interventions including semi-recumbent positioning and IOP-lowering medications, and anterior chamber paracentesis prepared for IOP >35 mmHg.

Patients were specifically questioned about symptoms, including pain, vision changes, discharge, or any concerns. A standardized adverse event reporting form was used to document all complications, regardless of severity. Late-onset complications (occurring >48 h post-procedure) were specifically tracked to identify any delayed safety issues that might be missed in traditional same-day discharge protocols.

#### 2.7.5 Patient satisfaction assessment

To address potential cultural and reporting bias, several measures were implemented: (1) the satisfaction survey was distributed and collected anonymously by trained specialized nurses at patient discharge, (2) patients were explicitly assured that their responses would remain confidential and would not affect their future care, (3) patients were encouraged to provide honest feedback regardless of cultural background or perceived expectations about medical authority, and (4) the questionnaire included both quantitative ratings and an open-ended question: “Please describe your overall psychological experience with our healthcare service, including any concerns about emotional support and communication during your treatment.” The complete questionnaire is provided as Supplementary File 1.

### 2.8 Statistical analysis

All statistical analyses were performed using SPSS 29.0. We hypothesized that the integrated medical-nursing service model would improve patient satisfaction compared to conventional care. Medication compliance, standardized treatment rates, NEI-VFQ-25 scores, and complication rates were defined as primary outcomes, while patient satisfaction was a secondary outcome. Normally distributed variables (age, NEI-VFQ-25) were expressed as mean ± standard deviation and compared using independent t-tests. Non-normally distributed variables were presented as median (interquartile range) and analyzed using Mann-Whitney U tests. Categorical variables were presented as frequencies and percentages and compared using chi-square tests. For primary outcomes, multivariate logistic regression was performed to adjust for age, gender, diagnosis type, and disease duration. For the secondary outcome, Bonferroni correction was applied (α = 0.0125). Model goodness-of-fit was assessed using the Hosmer-Lemeshow test. Statistical significance was set at *P* < 0.05 (two-sided) for the primary outcome and *P* < 0.0125 (two-sided) for secondary outcomes.

### 2.9 Ethical considerations

This retrospective cohort study was approved by the Clinical Trial Ethics Committee of the Affiliated Hospital of Southwest Medical University (approval number: KY2025127).

## 3 Results

### 3.1 Population baseline characteristics

A total of 1,432 patients were included in the analysis, with 631 in the control group (2023) and 801 in the intervention group (2024). Baseline characteristics are presented in Table 2. No statistically significant differences were observed between groups for any measured characteristic. The mean age was 59.22 ± 13.24 years in the control group versus 58.33 ± 13.33 years in the intervention group (mean difference = 0.89 years, 95% CI: −2.28 to 0.50, *P* = 0.210). Gender distribution was similar between groups (46.4% vs. 46.9% male, *P* = 0.849). Disease type distribution showed no significant difference (*P* = 0.191), with diabetic macular edema being the most common diagnosis in both groups. The balanced baseline characteristics confirm the comparability of study groups and support the validity of subsequent comparisons.

**Table 2:** Baseline characteristics of control and intervention groups.

**Characteristics**	**Control group (*n* = 631)**	**Intervention group (*n* = 801)**	**Statistics**	***P* value**	**95%CI**
Age (years), mean ± SD	59.22 ± 13.24	58.33 ± 13.33	1.255	0.210^a^	−0.50–2.28
**Gender**, ***n*** **(%)**					
Male	293 (46.40)	376 (46.90)	0.036	0.849^b^	
Female	338 (53.60)	425 (53.10)			
**Disease Type**, ***n*** **(%)**					
DME	252 (39.90)	358 (44.70)	4.748	0.191^b^	
AMD	126 (20.00)	160 (20.00)			
RVO, ME	161 (25.50)	190 (23.70)			
CNV	92 (14.60)	93 (11.60)			

^*a*^P-values were calculated with the T-test. ^*b*^P-values were calculated with the chi-square test. DME diabetic macular edema, AMD age-related macular degeneration, RVO retinal vein occlusion, ME macular edema, CNV choroidal neovascularization, SD standard deviation, CI confidence interval.

### 3.2 Medication compliance and treatment standardization

The intervention group showed markedly higher correct usage rates of preoperative antibiotic eye drops (98.0% vs. 82.6%, OR = 10.36, 95% CI: 6.06–17.70, *P* < 0.001), with an absolute risk reduction of 15.4% (95% CI: 12.8–18.1%) and NNT of 6. Similarly, standardized treatment rates (defined as ≥3 intravitreal injections) were significantly higher in the intervention group (43.6% vs. 25.2%, OR = 2.29, 95% CI: 1.83–2.88, *P* < 0.001), representing an 18.4% absolute improvement (95% CI: 14.0–22.7%) with NNT of 5 (Table 3).

**Table 3:** Medication compliance, treatment standardization, and quality of life.

**Outcome**	**Control group (*n* = 631)**	**Intervention group (*n* = 801)**	**OR (95%CI)**	**Statistics**	***P* value**	**ARR (95%CI)**	**NNT**
**Usage**							
Corrent, *n* (%)	521 (82.6)	785 (98.0)	10.36 (6.06–17.70)	104.788	< 0.001	15.4% (12.8–18.1)	6
**Standardized treatment**							
≥3 injections, *n* (%)	159 (25.2)	349 (43.6)	2.29 (1.83–2.88)	52.048	< 0.001	18.4% (14.0–22.7)	5
NEI-VFQ-25 score, mean ± SD	61.7 ± 13.8	79.8 ± 10.9		27.852	< 0.001		
Mean difference (95% CI)			18.2 (16.9–19.4)				
Effect size (Cohen's *d*)			1.48 (large)				

OR odds ratio, CI confidence interval, ARR absolute risk reduction, NNT number needed to treat, NEI-VFQ-25 National Eye Institute Visual Function Questionnaire-25, SD standard deviation, Bonferroni-corrected significance level: P < 0.0125.

### 3.3 Visual function-related quality of life

The intervention group demonstrated significantly higher NEI-VFQ-25 composite scores (79.8 ± 10.9 vs. 61.7 ± 13.8, mean difference = 18.2, 95% CI: 16.9–19.4, *P* < 0.001). The effect size was large (Cohen's *d* = 1.48), indicating clinically meaningful improvement in visual function-related quality of life beyond statistical significance (Table 3).

### 3.4 Postoperative complications analysis with severity grading

Postoperative complications were systematically classified according to predefined severity criteria (Table 4A) and analyzed with detailed subgroup analysis (Table 4B). The intervention group experienced significantly lower rates of all measured complications across all severity levels.

**Table 4A:** Complications severity grading criteria.

**Complication type**	**Severity grade**	**Clinical criteria**	**Management requirements**
Subconjunctival hemorrhage	Mild	< 25% of conjunctival surface	Observation only
	Moderate	25–50% of conjunctival surface	Symptomatic treatment
	Severe	>50% of conjunctival surface	Close monitoring, possible intervention
Postoperative IOP elevation	Mild	IOP < 30 mmHg	No treatment required, transient elevation
	Moderate	30–40 mmHg	IOP-lowering medications required
	Severe	IOP > 40 mmHg	Multiple medications or anterior chamber paracentesis
Infectious conjunctivitis	Mild	Minimal discharge, mild hyperemia	Topical antibiotics
	Moderate	Moderate purulent discharge, significant hyperemia	Intensive topical therapy
	Severe	Severe purulent discharge, marked hyperemia ± corneal involvement	Systemic antibiotics consideration
Endophthalmitis	Mild	Suspected, minimal symptoms	Close monitoring, culture
	Moderate	Confirmed, moderate vitritis	Intravitreal antibiotics
	Severe	Severe vitritis, vision threatening	Vitrectomy + antibiotics
Retinal detachment	Mild	Localized, asymptomatic	Observation, positioning
	Moderate	Involving macula periphery	Urgent surgical repair
	Severe	Involving central macula	Emergency surgical repair

IOP: Intraocular pressure.

**Table 4B:** Postoperative complications analysis with severity grading.

**Complication type**	**Control group (*n* = 631)**	**Intervention group (*n* = 801)**	**OR (95%CI)**	**Statistics**	***P* value**	**ARR (95%CI)**	**NNT**
**Subconjunctival hemorrhage, n (%)**							
Mild	22 (3.49)	6 (0.75)	0.21 (0.08–0.52)	18.647	< 0.001	2.74% (1.45%−4.03%)	36
Moderate	12 (1.90)	3 (0.37)	0.19 (0.05–0.70)	7.892	0.005	1.53% (0.46%−2.60%)	65
Severe	2 (0.32)	0 (0.00)			0.111	0.32% (−0.12–0.76%)	313
Total	36 (5.71)	9 (1.12)	0.19 (0.09–0.39)	24.340	< 0.001	4.59% (2.84–6.34%)	22
**Postoperative IOP elevation**							
Mild	12 (1.90)	1 (0.12)	0.06 (0.01–0.49)	7.892	0.005	1.78% (0.55%−3.01%)	56
Moderate	8 (1.27)	1 (0.12)	0.10 (0.01–0.78)	5.148	0.023	1.15% (0.16%−2.14%)	87
Severe	1 (0.16)	0 (0.00)			0.260	0.16% (−0.15%−0.47%)	631
Total	21 (3.33)	2 (0.25)	0.07 (0.02–0.31)	21.260	< 0.001	3.08% (1.70%−4.46%)	32
**Infectious conjunctivitis**							
Mild	11 (1.74)	4 (0.50)	0.28 (0.09–0.89)	4.498	0.034	1.24% (0.09%−2.39%)	81
Moderate	8 (1.27)	0 (0.00)			0.001	1.27% (0.55%−1.99%)	79
Severe	4 (0.63)	0 (0.00)			0.024	0.63% (0.08%−1.18%)	158
Total	23 (3.65)	4 (0.50)	0.13 (0.05–0.38)	18.880	< 0.001	3.15% (1.069%−4.61%)	32
**Endophthalmitis**							
Mild	0 (0.00)	0 (0.00)					
Moderate	0 (0.00)	0 (0.00)					
Severe	0 (0.00)	0 (0.00)					
Total	0 (0.00)	0 (0.00)					
**Retinal detachment**							
Mild	0 (0.00)	0 (0.00)					
Moderate	0 (0.00)	0 (0.00)					
Severe	0 (0.00)	0 (0.00)					
Total	0 (0.00)	0 (0.00)					
Overall complications	80 (12.68)	15 (1.87)	0.13 (0.07–0.23)	64.425	< 0.001	10.81% (8.35%−13.27%)	9

#### 3.4.1 Subconjunctival hemorrhage

The overall incidence of subconjunctival hemorrhage was markedly reduced in the intervention group (1.12% vs. 5.71%, OR = 0.19, 95% CI: 0.09–0.39, *P* < 0.001). This represented an absolute risk reduction of 4.59% (95% CI: 2.84–6.34%) with a number needed to treat of 22. The reduction was consistent across all severity grades: mild cases in patients decreased from 3.49% to 0.75% (ARR = 2.74%, NNT = 36), moderate patients from 1.90% to 0.37% (ARR = 1.53%, NNT = 65), and severe cases from 0.32% to 0% in the intervention group.

#### 3.4.2 Postoperative intraocular pressure elevation

Postoperative IOP elevation showed similar patterns of improvement, with overall incidence reduced from 3.33% in the control group to 0.25% in the intervention group (OR = 0.07, 95% CI: 0.02–0.31, *P* < 0.001). The absolute risk reduction was 3.08% (95% CI: 1.70–4.46%) with NNT of 32. The improvement was most pronounced in mild (1.90% vs. 0.12%) and moderate cases (1.27% vs. 0.12%), with no severe cases requiring multiple medications or paracentesis in the intervention group.

#### 3.4.3 Infectious conjunctivitis

The intervention group experienced significantly lower rates of infectious conjunctivitis (0.50% vs. 3.65%, OR = 0.13, 95% CI: 0.05–0.38, *P* < 0.001), with absolute risk reduction of 3.15% (95% CI: 1.069–4.61%) and NNT of 32. Notably, no moderate or severe complications in patients requiring intensive therapy or systemic antibiotics occurred in the intervention group, compared to eight moderate and four severe cases in the control group.

#### 3.4.4 Overall complication rates

When considering all complications combined, the intervention group had dramatically lower overall complication rates (1.87% vs. 12.68%, OR = 0.13, 95% CI: 0.07–0.23, *P* < 0.001), representing an absolute risk reduction of 10.81% (95% CI: 8.35–13.27%) and NNT of 9. No cases of endophthalmitis or retinal detachment were observed in either group during the study period.

### 3.5 Patient satisfaction with nursing care

Table 5 presents the comparison of patient satisfaction between groups. The intervention group demonstrated significantly higher overall satisfaction compared to the control group (99.0% vs. 89.4%, OR = 11.78, 95% CI: 5.61–24.71, *χ*^2^ = 65.804, *P* < 0.001). Specifically, 73.0% of intervention group patients reported being “very satisfied” compared to 31.1% in the control group. The absolute risk difference was 9.6% (95% CI: 7.1–12.1), indicating a number needed to treat (NNT) of 10 (95% CI: 8–14). The effect size was large (Cramér's *V* = 0.29), indicating substantial clinical impact beyond statistical significance.

**Table 5:** Patient satisfaction.

**Satisfaction level**	**Control group (*n* = 631)**	**Intervention group (*n* = 801)**	**Statistics**	***P* value**	**OR (95% CI)**
Very satisfied, *n* (%)	196 (31.06)	585 (73.03)			
Satisfied, *n* (%)	368 (58.32)	208 (25.96)			
Unsatisfied, *n* (%)	67 (10.61)	8 (0.99)			
Overall satisfaction, *n* (%)	89.38	98.99	65.804	< 0.001^a^	11.78 (5.61–24.71)

*a*P-values were calculated with the chi-square test.

Effect measures: Absolute risk difference 9.6% (95% CI 7.1 to 12.1); Number needed to treat 10 (95% CI 8 to 14); Cramér's V = 0.29 (large effect).

OR odds ratio, CI confidence interval. Overall satisfaction includes very satisfied and satisfied responses.

### 3.6 Multivariable analysis of patient satisfaction

Multivariable logistic regression analysis was performed to assess the independent effect of the intervention while adjusting for potential confounders (Table 6). After adjustment for age, gender, and disease type, the intervention group remained significantly associated with higher patient satisfaction (Adjusted OR = 11.31, 95% CI: 5.39–23.72, *P* < 0.001). The model demonstrated good fit (Hosmer-Lemeshow test: *χ*^2^ = 8.34, *P* = 0.400) and moderate predictive ability (Nagelkerke *R*^2^ = 0.156; Area under ROC curve = 0.723, 95% CI: 0.686–0.759).

**Table 6:** Multivariable logistic regression analysis for patient satisfaction.

**Variable**	**Crude OR (95%CI)**	***P* value**	**Adjusted OR (95%CI)**	***P* value**
**Treatment group**				
Control	1 (Reference)		1 (Reference)	
Intervention	11.78 (5.61–24.71)	<0.001	11.31 (5.39–23.72)	<0.001
**Age(years)**	0.99 (0.98–1.00)	0.156	0.99 (0.98–1.00)	0.198
**Gender**				
Female	1 (Reference)		1 (Reference)	
Male	1.12 (0.86–1.45)	0.394	1.08 (0.82–1.42)	0.586
**Disease type**				
DME	1 (Reference)		1 (Reference)	
AMD	0.94 (0.67–1.32)	0.724	0.91 (0.64–1.29)	0.596
RVO, ME	1.18 (0.84–1.66)	0.334	1.15 (0.81–1.63)	0.428
CNV	0.89 (0.58–1.37)	0.598	0.86 (0.55–1.34)	0.507

*Model performance*: Hosmer-Lemeshow test χ^2^ = 8.34, *P* = 0.400; Nagelkerke *R*^2^ = 0.156; Area under ROC curve = 0.723 (95% CI 0.686–0.759).

*OR* odds ratio, *CI* confidence interval, *DME* diabetic macular edema, *AMD* age-related macular degeneration, *RVO* retinal vein occlusion, *ME* macular edema, *CNV* choroidal neovascularization.

Model performance: Hosmer-Lemeshow test χ^2^ = 8.34, df = 8, *P* = 0.400; Nagelkerke *R*^2^ = 0.156; Area under ROC curve = 0.723 (95% CI: 0.686–0.759).

The minimal change between crude (OR = 11.78) and adjusted estimates (OR = 11.31) indicates that the observed improvements are primarily attributable to the intervention rather than baseline differences or confounding. Neither age (Adjusted OR = 0.99 per year, 95% CI: 0.98–1.00, *P* = 0.198), gender (Adjusted OR = 1.08 for males vs. females, 95% CI: 0.82–1.42, *P* = 0.586), nor disease type showed significant associations with patient satisfaction in the multivariable model, suggesting that the intervention effect is robust across patient subgroups.

## 4 .Discussion

### 4.1 Principal findings

Our study demonstrates that the medical-nursing integrated service model significantly improves multiple aspects of outpatient intravitreal injections procedures by addressing coordination challenges that persist even in established same-day service models. Key findings include higher patient satisfaction, improved compliance with preoperative antibiotic eye drops, increased standardized treatment rates, enhanced patient self-management efficacy, and reduced complication rates.

### 4.2 Improved treatment compliance and standardization

The significant improvement in the correct usage of preoperative antibiotic eye drops demonstrates how integrated care enhances treatment adherence. Research has shown that interprofessional collaborative practice significantly improves medication adherence and treatment outcomes across multiple healthcare settings (26). Before implementing the integrated model, doctors and nurses conveyed surgery-related knowledge separately, primarily through verbal instructions, which patients easily forgot. After implementation, medical staff provided comprehensive information and distributed outpatient procedure instruction sheets, particularly regarding antibiotic eye drop usage, guidance on controlling underlying diseases, and individualized anticoagulation management. For patients on anticoagulant therapy, management decisions followed our institutional protocol based on individual risk assessment, with continuation of anticoagulation for most patients unless specific contraindications existed, in alignment with current recommendations for low-risk ophthalmic procedures.

The markedly increased rate of standardized treatment (defined as receiving ≥3 intravitreal injections) suggests that the integrated model promotes better continuity of care. The established follow-up mechanisms facilitated ongoing patient engagement and likely reduced loss to follow-up. Studies have found that structured collaborative care models significantly improve treatment protocol adherence and continuity of care (27).

### 4.3 Improved vision-related quality of life

The medical-nursing integrated service model produced significant positive effects on patients' vision-related quality of life, which likely resulted from multiple factors working in concert. This integrated approach provided patients with comprehensive disease education and emotional support, addressing both the physical aspects of treatment and the psychological impact of visual impairment. Previous research has identified psychological well-being as a crucial factor affecting patients' quality of life (28–30). Concurrently, our findings align with previous research demonstrating that medical-nursing integrated service can significantly improve patients' quality of life (31).

The medical-nursing integrated service model increases patients' access to health knowledge while ensuring planned, continuous, and comprehensive health education. This meets the expectations of patients and their families for rehabilitation from their disease and fulfills their needs for health information (32). By better understanding treatment expectations, patients can participate more effectively in their care and support lifestyle modifications, thereby enhancing their quality of life.

### 4.4 Reduced complications

The intervention group experienced significantly lower rates of all measured complications (subconjunctival hemorrhage, postoperative intraocular pressure elevation, and infectious conjunctivitis), demonstrating the clinical benefit of the integrated approach. These improvements could be attributed to several factors, including better preoperative preparation, enhanced patient compliance with instructions, standardized procedures, and improved communication between healthcare providers. Previous studies have found that organized interprofessional collaboration through better care coordination and information transfer significantly reduces adverse events (33).

The systematic quality control measures implemented in the integrated model, including standardized documents for complication prevention and medical-nursing collaborative quality evaluation, created a more robust safety system. The model emphasizes comprehensive medical-nursing collaboration, fully leveraging medical staff's initiative, providing timely feedback, and continuous improvement. It enhances medical-nursing cooperation and work efficiency, strengthens patient and family compliance, and consequently reduces postoperative complications and improves medical care quality. This finding aligned with research conducted by Porras-González and collaborators in 2015, which showed that integrated nursing methodology in major ambulatory surgery led to significant reductions in surgical wound complication rates in outpatient procedure settings (34).

### 4.5 Enhanced patient satisfaction

The integrated medical-nursing service model substantially improved patient satisfaction compared with the traditional care model. This improvement can be attributed to several factors inherent in the integrated approach. The designated clinic room with specialized staff created a more focused and efficient care environment where both medical and nursing staff collaborated to address concerns comprehensively. Research has demonstrated that integrated care approaches improve patient satisfaction and clinical outcomes in ambulatory surgery settings (35).

Due to the short hospital stay during outpatient procedure, patients often face challenges in understanding relevant information in a limited time. The integrated model addresses this through comprehensive medical care services, strengthening guidance for patients throughout the entire process. Medical staff and nurses adopt a complementary approach to patient education, resulting in better knowledge retention and compliance. The WeChat platform further builds an integrated service model, allowing patients to receive timely solutions to their problems, thus improving overall satisfaction (36).

### 4.6 Limitations and future directions

This single-center retrospective study has several important limitations. Firstly, the non-randomized, sequential design introduces potential temporal confounding, though baseline characteristics were similar between groups. Secondly, the retrospective design limits control for unmeasured confounders such as socioeconomic status, health literacy, or patient motivation. Thirdly, the single-center design limits generalizability to other healthcare systems and patient populations. Fourthly, we did not conduct cost-effectiveness analysis or assess long-term outcomes beyond the immediate perioperative period. Fifthly, this study evaluated the integrated model as a whole package of multiple interventions, making it difficult to identify which specific elements were most responsible for the improvements observed. Sixthly, patient satisfaction assessment may be influenced by subjective factors, such as reluctance to express dissatisfaction due to respect for medical authority. Seventhly, unlike the 24-h hospitalization model with routine next-day physician examination, our integrated model relied on structured Day 1 telephone follow-up supplemented by mandatory Day 7 in-person evaluation. While complication detection rates showed no significant differences between groups, we acknowledge that physical examination remains the gold standard for clinical assessment. Eighthly, for geographically distant patients, same-day discharge may present transportation challenges and difficulties returning for scheduled follow-up visits; our institution maintained a 24-h observation unit for such cases, though this represents a hybrid approach that may limit the model's efficiency gains for this subpopulation. Our study did not evaluate potential integration with emerging healthcare delivery approaches, such as telemedicine-enhanced monitoring or expanded nursing roles in procedural care.

Despite these limitations, the substantial improvements observed across multiple outcome domains provide strong evidence for the intervention's effectiveness. Future research should address several areas to strengthen the evidence base. First, multicenter randomized controlled trials with concurrent control groups are recommended to address temporal confounding. Second, prospective studies with comprehensive baseline data collection would better control for confounders and strengthen causal inferences. Third, multi-center implementation studies across diverse healthcare settings are suggested to enhance generalizability. Fourth, future studies should incorporate economic evaluation and extended follow-up periods to assess sustainability of benefits. Fifth, future research should test each part of the integrated model separately to determine which specific parts are most effective in improving patient outcomes. Sixth, development of more objective patient satisfaction assessment tools is suggested to enhance reliability across different contexts. Seventh, future implementations should consider developing validated telemedicine protocols with standardized photo documentation to address follow-up challenges for geographically dispersed populations. Finally, integration with digital health technologies is recommended to enhance the model's effectiveness while addressing scalability concerns.

## 5 Conclusion

The medical-nursing integrated service model represented a significant advancement in outpatient ophthalmic care management, demonstrating improvements across multiple performance domains. Our findings suggested that this approach effectively addressed many traditional challenges in outpatient procedure delivery while enhancing both patient outcomes and operational efficiency. The success of this model in improving patient satisfaction, medication adherence, increasing standardized treatment rates, enhancing visual function-related quality of life, and reducing the incidence of postoperative complications provides strong evidence for its wider implementation. The evidence presented in this study strongly supported the wider adoption of integrated medical-nursing service models in ophthalmic outpatient procedures. The significant improvements in patient outcomes and operational efficiency suggested that this approach could help healthcare organizations meet the growing demands for efficient surgical care while maintaining high standards of patient safety and satisfaction.

## Data Availability

The raw data supporting the conclusions of this article will be made available by the authors, without undue reservation.
