# Supplementary material for: Medical-nursing integrated service model in ophthalmic outpatient care for intravitreal injections: a retrospective cohort study
Source: Front Med (Lausanne). 2025 Dec 16;12:1689049. doi: 10.3389/fmed.2025.1689049 (PMC12748181; doi:10.3389/fmed.2025.1689049)
Supplement: Supplementary file 1 [file Table_1.docx]

Supplementary Material

**Supplementary file 1:Patient Satisfaction Questionnaire for Intravitreal Injection Services**

**Instructions**

Please rate your satisfaction with each aspect of your care experience during your intravitreal injection treatment. Your responses are completely confidential and will not affect your future care. Please answer honestly based on your actual experience.

| **Evaluation Items** | **Very Satisfied (5)** | **Satisfied (4)** | **Neutral (3)** | **Dissatisfied (2)** | **Very Dissatisfied (1)** |
| --- | --- | --- | --- | --- | --- |
| **Communication Quality** | | | | | |
| 1. How satisfied were you with the clarity of explanations about your eye condition and intravitreal injection treatment? | | | | | |
|  | □ | □ | □ | □ | □ |
| 2. How satisfied were you with the medical staff's answers to your questions about intravitreal injection treatment? | | | | | |
|  | □ | □ | □ | □ | □ |
| 3. How satisfied were you with the nursing staff's communication during the injection treatment process? | | | | | |
|  | □ | □ | □ | □ | □ |
| 4. How satisfied were you with the consistency of information provided by different staff members? | | | | | |
|  | □ | □ | □ | □ | □ |
| **Care Coordination** | | | | | |
| 5. How satisfied were you with the coordination between doctors and nurses during your intravitreal injection treatment? | | | | | |
|  | □ | □ | □ | □ | □ |
| 6. How satisfied were you with the timeliness and convenience of scheduling your injection treatment? | | | | | |
|  | □ | □ | □ | □ | □ |
| 7. How satisfied were you with the smooth flow of your care from consultation to injection completion? | | | | | |
|  | □ | □ | □ | □ | □ |
| 8. How satisfied were you with the post-injection follow-up arrangements and guidance? | | | | | |
|  | □ | □ | □ | □ | □ |
| **Technical Competence** | | | | | |
| 9. How satisfied were you with the physician's professional skills in performing the intravitreal injection? | | | | | |
|  | □ | □ | □ | □ | □ |
| 10. How satisfied were you with the nursing staff's professional skills and assistance? | | | | | |
|  | □ | □ | □ | □ | □ |
| 11. How satisfied were you with the sterile techniques and safety measures during the injection procedure? | | | | | |
|  | □ | □ | □ | □ | □ |
| 12. How satisfied were you with the quality of medical instruments and medications used? | | | | | |
|  | □ | □ | □ | □ | □ |
| **Facility Environment** | | | | | |
| 13. How satisfied were you with the cleanliness and sterile environment of the injection treatment room? | | | | | |
|  | □ | □ | □ | □ | □ |
| 14. How satisfied were you with the comfort and privacy of the injection treatment environment? | | | | | |
|  | □ | □ | □ | □ | □ |
| 15. How satisfied were you with the layout and accessibility of the outpatient intravitreal injection area? | | | | | |
|  | □ | □ | □ | □ | □ |
| 16. How satisfied were you with the availability of educational materials about intravitreal injection treatment? | | | | | |
|  | □ | □ | □ | □ | □ |
| **Overall Experience** | | | | | |
| 17. How satisfied were you with the respect and courtesy shown by all staff members? | | | | | |
|  | □ | □ | □ | □ | □ |
| 18. How satisfied were you with the emotional support and care provided during treatment? | | | | | |
|  | □ | □ | □ | □ | □ |
| 19. How satisfied were you with the overall quality of medical and nursing care? | | | | | |
|  | □ | □ | □ | □ | □ |
| 20. How likely are you to recommend our intravitreal injection services to others with similar conditions? | | | | | |
|  | □ | □ | □ | □ | □ |

**Additional Comments**

**Please describe your overall experience with our intravitreal injection healthcare service, including any suggestions for improvement:**

**Thank you for your feedback!**
